# Thymol has beneficial effects on the experimental model of ulcerative colitis

**DOI:** 10.22038/AJP.2019.13383

**Published:** 2019

**Authors:** Pourya Tahmasebi, Seyyed Meysam Abtahi Froushani, Nahideh Afzale Ahangaran

**Affiliations:** 1 *Department of Microbiology, Faculty of Veterinary Medicine, Urmia University, Urmia, Iran*

**Keywords:** Thymol, Ulcerative colitis, Acetic-acid, Wistar rat

## Abstract

**Objective:**

Thymol, a natural aromatic monoterpene phenol derived from thymus, possesses anti-inflammatory benefits. Here, we evaluated the potential of thymol therapy in improving an animal model of ulcerative colitis.

**Materials and Methods:**

Luminal instillation of acetic acid was used to induce colitis in male Wistar rats. Treatment groups daily received prednisolone (2 mg/kg, orally) or thymol (100 mg/kg, orally) for 10 consecutive days. Then, the rats were euthanized and tissue specimens were collected for evaluation of cyclooxygenase-2 (COX-2) expression by immunohistochemistry. Furthermore, the levels of total protein, nitric oxide, myeloperoxidase, malondialdehyde, IL-1, IL-6, and TNF-α were monitored in colonic homogenates. Eventually, the relative mRNA expression of *IκBα *and *NF-κBp65* was investigated using reverse-transcriptase PCR (RT-PCR) in colonic homogenates.

**Results:**

Both medications could reduce the mortality rate and the clinical scores of ulcerative colitis. The COX-2 expression was significantly reduced in the colons of thymol-treated animals compared to the prednisolone group. Also, the myeloperoxidase activity, nitric oxide level and malondialdehyde intensity were decreased in the colons of thymol-treated animals to a greater extent compared to the prednisolone group. Moreover, the total protein content of guts showed significant increases in the guts of thymol-treated animals in comparison to the prednisolone group. Nonetheless, thymol significantly reduced the levels of IL-6, and IL-1 compared to prednisolone. Both medications caused a significant decrease in the mRNA level of *NF-κBp65*, though the mRNA level of *IκBα* did not show significant changes between the groups.

**Conclusion:**

Thymol may be a promising agent to ameliorate ulcerative colitis.

## Introduction

Ulcerative colitis (UC) is one of the principal subtypes of inflammatory bowel syndrome (IBD), affecting mostly the rectum and left colon (Cho, 2008; Hindryckx et al., 2016). Increasing rate of prevalence and incidence of inflammatory bowel disease (IBD) indicate its emergence as a global disease (Molodecky et al., 2012). Although the pathogenesis of IBD in dogs as well as humans has not been known well, there is a consensus that polymorphonuclear leukocyte infiltration, reactive oxygen species (ROS), lipid mediators as well as metabolites of the arachidonic acid such as leukotrienes and prostaglandins play a crucial role in the development of inflammation in IBD (Gorgulu et al., 2006; Hawes et al., 2018; Keshavarzian et al., 1990; Millar et al., 1996). Hence, first-line therapy for IBDs is common anti-inflammatory drugs, including corticosteroids and aminosalicylates (like Mesalazine, Sulfasalazine, Olsalazine, and Balsalazide); however despite their high efficiency, these drugs have considerable side effects such as skin thinning, ophthalmic disorders, infections, nausea, indigestion, headache, vomiting, abdominal pain, diarrhea, and flatulence (D'haens, 2016; Hauso et al., 2015; Hindryckx et al., 2016; Wang et al., 2016). Nevertheless, the need for a safer and less risky therapeutic strategy for patients with IBD is felt.

A fundamental advance in the investigation of IBD has been experimental-animal models that permitting the investigation of early events, evaluation of the interactions among various therapeutic components, and investigation of immunologic processes (Blumberg et al., 1999; Elson et al., 1995). The results obtained with experimental models will contribute to better design of preclinical study to target particular components involved in the pathogenesis of IBD (Kim and Berstad, 1992; Low et al., 2013). To evaluate anti-inflammatory properties and free radical scavenging capacity, male Wistar rats were induced with %5 acetic acid intraluminal. Acetic-acid induced ulcerative colitis considerably resembles the pattern of arachidonate metabolism and histopathological alteration that is identical to human UC (Low et al., 2013; Strober, 1985). Nowadays, medicinal herbs with anti-inflammatory immunomodulatory properties and fewer side effects can offer a new insight into the folk medicine (Abtahi Froushani et al., 2016). Thymol is a famous natural aromatic monoterpene phenol and found and abundantly in plants belonging to the Lamiaceae family (thymus,* Monarda genera,* ocimum and origanum) and many other medicinal plants (Marchese et al., 2016; Ribeiro et al., 2016). Thymol has been considerable with many pharmacological potential such as anti-inflammatory benefits (Fachini-Queiroz et al., 2012; Liang et al., 2014a), cicatrizing and wound healing (Marchese et al., 2016) (Riella et al., 2012), as well as insecticidal (Tang et al., 2011), antifungal (Ahmad et al., 2011) and antioxidant properties (Yu et al., 2016). Healing benefits of thymol on acute and chronic gastric ulcers have been reported in rats (Ribeiro et al., 2016). Nevertheless, there are no or limited data on the potential benefits of thymol; on the autoinflammatory condition like UC. Therefore, the present survey was applied to assess the potential role of thymol against the ulcerative colitis induced by acetic acid in Wistar rats.

## Materials and Methods

**Reagents**

Prednisolone was purchased from Aburaihan Pharmaceutical Company. (Tehran, Iran). The enzyme-linked immunosorbent assay (ELISA) kits were provided from PeproTech EC, Ltd. (London, UK). Total protein assay kit was procured from Zist-chemi Company. (Tehran, Iran). Thymol and other reagents were purchased from Sigma-Aldrich Corporation (St. Louis, MO, USA).

**Rats**

The male Wister rats (weighing 280-300 grams) were kept under constant environmental conditions, including a 12-hour light/dark cycle and 25°C temperature). Animals were fed *ad libitum* with standard laboratory chow and water. Ethical considerations were observed in concurrence with the guidelines of the Ministry of Health and Medical Education Guide for the Care and Use of Laboratory Animals.

**Induction of colitis** **and evaluation**

The animals were lightly anesthetized with ether after 48 hours of fasting. Then, the animals were intra-rectally instilled with 4% acetic acid (2 ml solution for each animal) using a rubber cannula (8 cm long). The animals were kept in that position for 20 sec, and before the withdrawal of the catheter, 5.0 ml of saline was flushed to ensure that the acetic acid diffused completely within the colon (Low et al., 2013). The Wistar rats were stochastically divided into the subsequent classes (n=10): vehicle-treated colitis rats, thymol-treated colitis rats (100 m/kg, per os-daily), prednisolone-treated colitis rats (4 mg/kg, per os-daily), and normal control rats. The control rats were intra-rectally consummated distilled water. The vehicle-treated colitis rats and normal rats were treated with vehicle (0.5 ml of PBS, PO, daily). 

Stool consistency, gross bleeding, and body weight were checked daily. The index of disease activity (DAI) was defined as the sum of grades of stool consistency, bloody feces, and weight loss pursuant to the properties determined in Table 1. The animals’ survivability was monitored daily within the survey. When a rat developed a DAI more than 8, the animal was euthanized a sampled for further experiments. The colon specimens were cut from 10 cm distal part of colon portions, and used for macroscopic evaluations (ulcer formation and hemorrhage).

**Homogenization of colonic samples**

The distal colons obtained from each group were weighed. The same amount of specimen was dissected, unpacked and homogenized in a frigid receptacle at a concentration of 10% (w/v) in 11.5 g/L dilution of KCl. The homogenized colon specimens were centrifuged at 10,000 *g* at 4°C for 10 minutes (Al-Rejaie et al., 2013a). 

**Table1:** Scoring system for evaluation the severity of ulcerative colitis (Abtahi Froushani and Mashouri 2018)

**Score**	**Weight loss **	**Stool consistency**	**Blood feces**
0	Negative	Normal	Negative
1	1-9%	Soft	Red
2	10-19%	Very Soft	Dark Red
3	<20%	Diarrhea	Black

The disease activity index (DAI) was reported as the sum of scores of all values.

**Activity of** **myeloperoxidase** **in colonic homogenates**

The activity myeloperoxidase (MPO) in the colonic specimens, was investigated according to the method as qualified previously (Pulli et al., 2013). Briefly, 10 µl of homogenized colon tissues were combined with 110 µl TMB solution (2.9 mM TMB in 14.5% DMSO plus 150 mM sodium phosphate buffer at pH 5.4) and 80 µl of 0.75 mM hydrogen peroxide. The specimen was kept for 5 minutes at 37°C. Afterward, to terminate the reaction, 50 µl of 2 M Sulfuric acid was mixed and the optical density was recorded at 450 nm. 10 µl of horseradish peroxidase (HRP) were applied as standard (2.5 and 25 milliunit/ml HRP). The activity of MPO was determined as the difference of the optical density relating to the standard curve of HRP. The findings were reported as milliunits per milliliter (mU/ml).

**Intensity of nitric oxide production in** **the colonic specimen**

The intensity of nitric oxide production in the gut specimen was monitored using the Griess assay procedure. 50 µl Griess reagent (3% phosphoric acid, 0.1% naphthyl ethylenediamine and 0.1% sulphanilamide) were mixed with 50 µl of homogenized tissue specimen and incubated for 10 minutes in the dark at room temperature. Afterward, the optical density was read at 540 nm using a standard microplate reader. Finally, a standard curve was drawn to estimate the level of nitrite (Bryan and Grisham, 2007).

**Estimation of malondialdehyde** **(MDA) in the specimens of colon**

The intensity of malondialdehyde level in the specimens of colon was checked as described earlier (Al-Rejaie et al., 2013b). Briefly, 2.5 ml reaction solution (0.25 M HCl, 15% trichloroacetic acid and 0.37% thiobarbituric acid, 1:1:1 ratio) was combined whith 100 µl of tissue homogenate and warmed for 60 minutes at 95°C. After cooling, the blend was centrifuged at 3500 g for 15 minutes. Eventually, the optical density of the isolated supernatant was recorded at 540 nm. The values were presented as nM of MDA/mg protein.

**Determination of pro-inflammatory cytokines in the colon specimens**

IL-6, IL-1β, and TNF-α levels were assessed in the homogenate of the colon specimens by using the commercial ELISA kits according to the manufacturer’s instructions (Gao et al., 2016).

**Estimation of total protein levels in the colon homogenate**

The protein content of the homogenized tissues was analyzed by using pyrogallol red-molybdate procedure in accordance whith the instructor’s guidelines (Orsonneau et al., 1989).

**COX-2 immunohistochemical staining**

IHC staining was followed using the standard method reported earlier (Xu et al., 2005).The slides of the tissue section were pre-warmed for 30 minuts at 60°C in a hot air oven. Xylene was used to de-paraffinize sections. The slides were rehydrated by an alcohol gradient (90%, 80%, 70%, and 50%). Afterward, 10 mM sodium citrate buffer was used for the antigen retrieval. Immunohistochemical staining method was implemented according to the producer’s guidelines (Biocare and ScyTek, USA). At first, to block the endogenous peroxidase, the specimens were rinsed for 5 min in a peroxidase blocking solution. This solution contained 0.03% hydrogen peroxide in sodium acid. The slides were leniently rinsed and subsequently stained overnight with COX-2 (1:600) primary antibody. Afterward, the sections were incubated with streptavidin conjugated to horseradish peroxidase (streptavidin–HRP) in a humidified chamber for 15 minutes. Next, the sections were washed, and a DAB chromogen was surcharged to the slides. After 5 minutes, the sides were stained with hematoxylin. Finally, the sides were fallen in a weak ammonia solution for 10 times, rinsed and cover slipped. The count of COX-2 positive cells was determined in one mm^2^ of the colonic tissues.

**mRNA expression of IκBα and NF-κB p65** **in the colonic specimens**

mRNA content of colon part of gut tissue was extracted using a commercial kit pursuant to the constructor 's guidelines. In brief, after synthesizing cDNA, a real-time reverse transcription PCR method was used for quantification. The subsequent sense and anti-sense sets were: PCR forward primer for NF-κB p65: 5′-TGCAGGCTCCTGTGCGAGTG-3′ and PCR reverse primer for NF-κB p65: 5′-TCCGGTGGCGATCGTCTGTGT-3′; PCR forward primer for IκBα: 5′-CGTGTCTGCACCTAGCCTCTATC-3′ and PCR forward reverse for IκBα: 5′-GCGAAACCAGGTCAGGATTC-3′ and the PCR forward primer for β-actin: 5′-GCAGGAGTACGATGAGTCCG-3′ and PCR reverse primer for β-actin: 5′-ACGCAGCTCAGTAACAGT CC-3′. The PCR schedule contained for 30 s at 95°C and 40 PCR cycles (5 sec at 95°C and 30 sec at 60°C). The β-actin was used for normalization of mRNA expression.

**Statistical surveys**

The nonparametric data (disease activity index), were assessed using the Kruskal Wallis exam conformed by Mann Whitney U evaluation with Bonferroni adjustment. The rest of the parametric values were analyzed, by the one-way ANOVA, in addition to Dunnett’s post hoc test. The survival function of lifetime results was evaluated by the Kaplan–Meier estimator. The findings were reported as means±SEM. The p-values less than 0.05 were recorded as statistically significant. 

## Results

**Disease activity index (DAI) findings**

**Figure 1:**
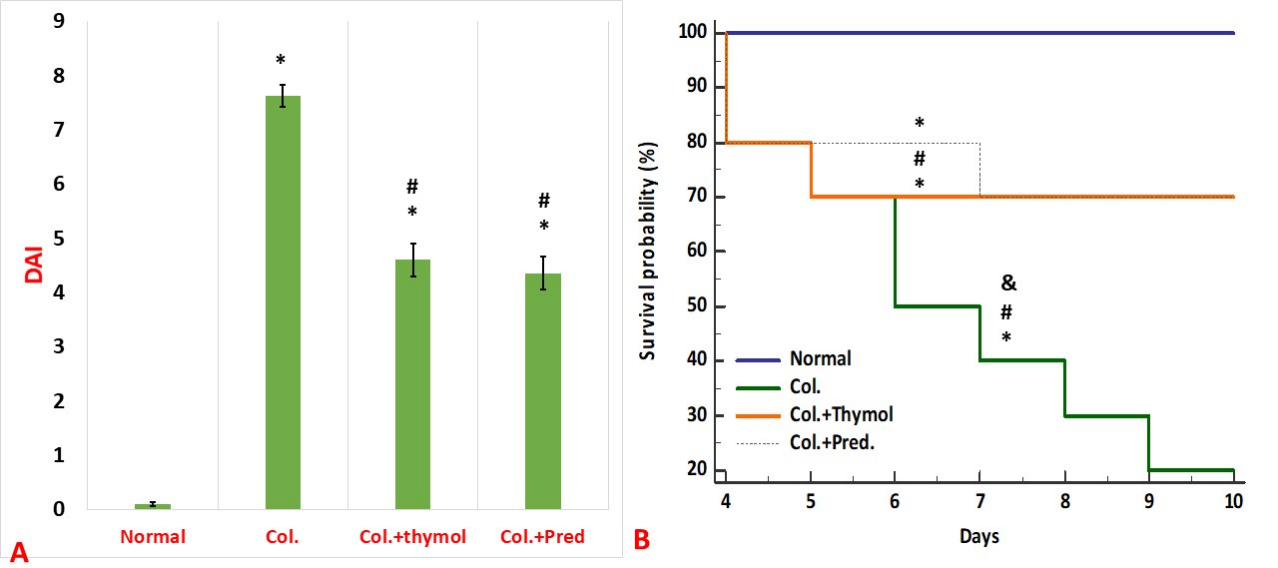
Assessment of disease activity index (A) and survival rate (B). The obtained data demonstrated that both thymol and prednisolone could ameliorate disease score and decrease the mortality rate of rats with ulcerative colitis in a comparable manner. Data were reported as mean±SEM (*p<0.05 versus normal control rats; ^#^p<0.05 versus vehicle- treated colitis group; ^&^p<0.051 versus thymol- treated colitis group). Col. Colitis; Pred.: Prednisolone

The weight and well-being of the rats were monitored daily after acetic acid instillation into the colonic lumen of the studied animals. According to Figure 1, induction of ulcerative colitis, led to a high mortality rate and a high DAI score in living rats. The data indicated that both medications could reduce the mortality rate and the clinical scores of ulcerative colitis in a similar scheme (Figure 1). As Figure 1 B displays, all control positive animals perished within 7 days after colitis induction, however, there were no deaths among normal rats in this course. The survival rate within ten days of the survey in both of the thymol and prednisolone treated groups was 70% (Figure 1 B). 

**Calculation of pro-inflammatory cytokine levels**

**Figure 2:**
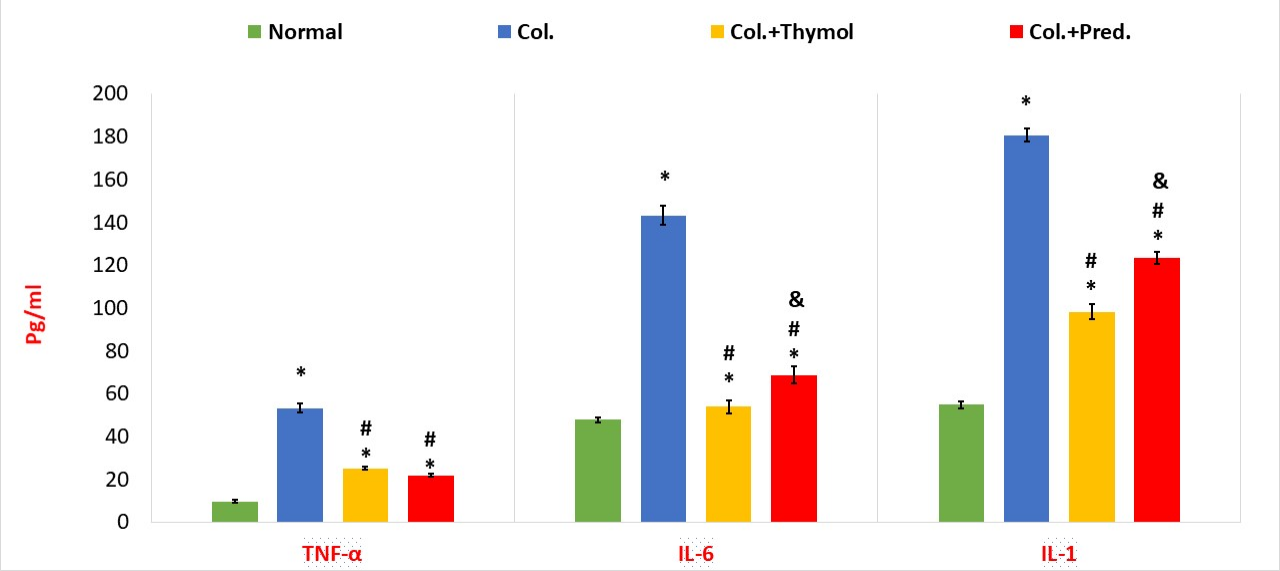
Evaluation of cytokine profile in the colonic specimens. The content of TNF-α, IL-1β, and IL-6 in colonic homogenates was markedly regressed in both treatment groups compared to colitis rats. Albeit, thymol significantly decreased the levels of IL-1 and IL-6 more than prednisolone. Values were reported as mean±SEM (*p<0.05 versus normal control rats; ^#^p<0.05 versus vehicle- treated colitis group; ^&^p<0.051 versus thymol- treated colitis group (. Col. Colitis; Pred.: Prednisolone

 As Figure 2 shows, the content of the IL-6, IL-1β and TNF-α in the colonic tissues was significantly enhanced in colitis rats compared to normal rats. Compared to the vehicle-treated colitis rats, both medications promoted a significant diminish in the content of these cytokines in the colonic specimens of rats with colitis (Figure 2). In addition, thymol significantly lessened the levels of IL-1 and IL-6 more profound than prednisolone (Figure 2). Moreover, there was no significant discernment in TNF-α levels between colitis rats treated with prednisolone or thymol (Figure 2). 

**Calculation of biochemical parameters**

**Figure 3:**
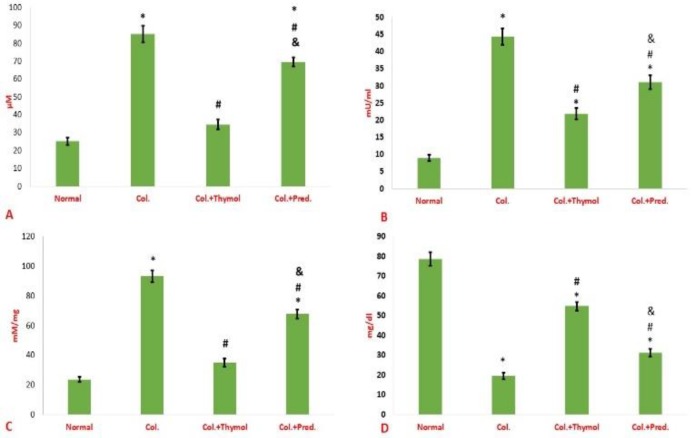
Biochemical changes in the colonic tissues. The levels of nitric oxide (A), myeloperoxidase activity (B) and malondialdehyde (C) were diminished in the guts of thymol treated rats more than prednisolone groups. Furthermore, both medication could equally increase the level of total protein in the colonic specimens compared to control positive rats (D). Data were reported as mean±SEM (*p<0.05 versus normal control rats; ^#^p<0.05 versus vehicle- treated colitis group; ^&^p<0.05 versus thymol- treated colitis group). Col. Colitis; Pred.: Prednisolone

In the following, the biochemical changes in the colonic tissues of the study population were evaluated. The obtained data indicated that the MPO activity and the malondialdehyde intensity as well as nitric oxide in the colonic homogenate of rats with colitis were signifacntly increased compared to normal rats (Figure 3 A-C). Also, total protein content of the colonic sample of colitis rats was significantly lessened compared to normal rats (Figure 3 D). Both medications induced a significant reduction in the activity of MPO and the intensity of nitric oxide production and malondialdehyde in the gut homogenate of animals with colitis compared to positive control animals (Figure 3 A-C). It should be noted that, the myeloperoxidase activity, nitric oxide level and malondialdehyde intensity were decreased in the colons of thymol treated studied animals more pronouncedly than prednisolone treated groups (Figure 3 A-C). Finally, the total protein content of colonic specimens was significantly increased in both treatment groups compared to control positive rats (Figure 3 D).

**COX-2 immunohistochemical staining**

**Figure 4:**
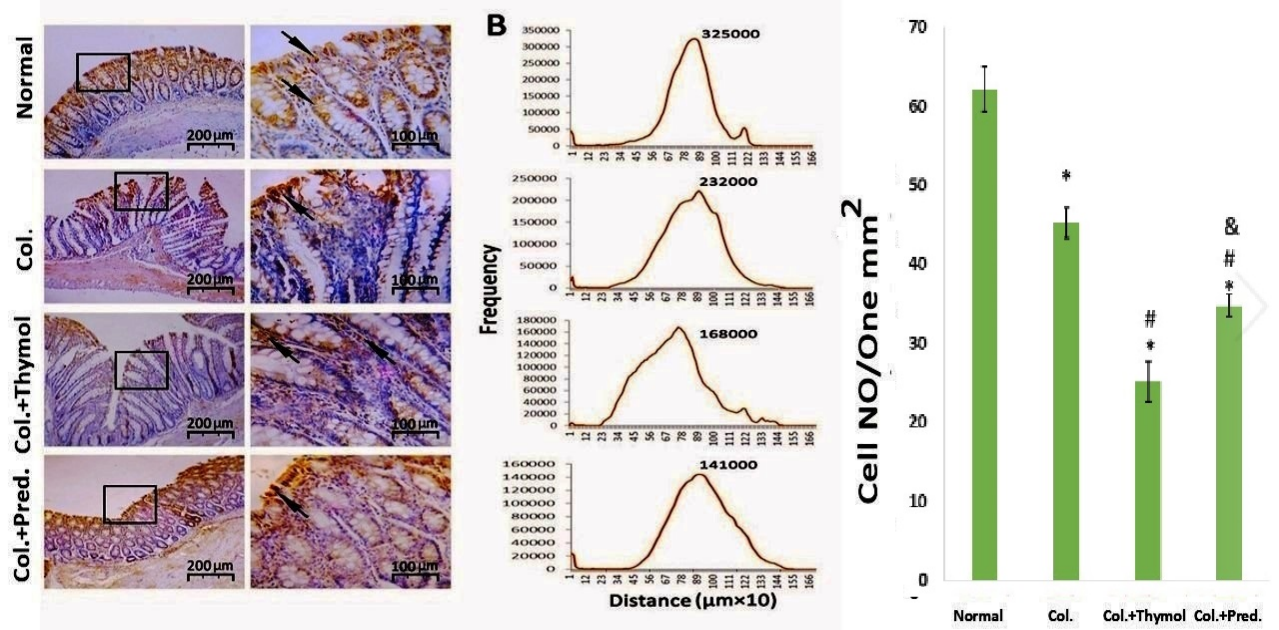
Immunohistochemical evaluation of the colonic tissues. The expression COX-2 was significantly decreased in the guts of thymol treated rats more than prednisolone groups. Data were expressed as mean±SEM (*p<0.05 versus normal control rats; ^#^p<0.05 versus vehicle- treated colitis group; ^&^p<0.05 versus thymol- treated colitis group). Col. Colitis; Pred.: Prednisolone

Immunohistochemical examination indicated that the expression of COX-2 was significantly increased in the colonic specimens of colitis rats compared to healthy rats (Figure 4). In this regard, the levels of COX-2 were significantly reduced in the guts of thymol treated rats more pronouncedly than prednisolone treated groups (Figure 4).

**Findings of reverse transcription polymerase chain reaction (RT-PCR)**

**Figure 5:**
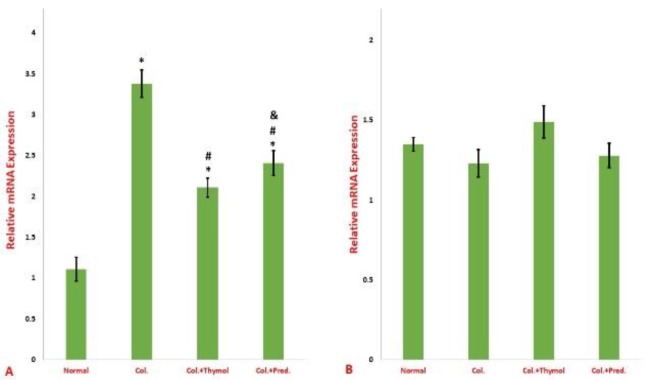
Relative mRNA expression of NF-κBp65 (A) and IκB (B) in gut tissues. The expression NF-κBp65 was significantly decreased in the guts of the both treatment groups compared to the control positive group. The expression of IκB didn’t show any significant difference between the groups. Results were reported as mean±SEM (*p<0.05 versus normal control rats; ^#^p<0.05 versus vehicle- treated colitis group; ^&^p<0.05 versus thymol- treated colitis group). Col. Colitis; Pred.: Prednisolone

As figure 5 shows, the level of NF-κBp65 mRNA in the colonic specimens was significantly increased in rats received intra-rectally acetic acid compared to normal rats. Both medications result in a significant regress in the mRNA level of NF-κBp65 (Figure 5). Furthermore, the analysis of the mRNA level of IκBα did not express any statistical significant diversity between the groups (Figure 5). 

## Discussion

The data of this investigation supported that both of prednisolone and thymol could similarly reduce the mortality rate and clinical scores of the animal model of ulcerative colitis. Instillation of acetic acid into the colon have been extensively used to evaluate the beneficial effects of new candidate medication to control UC (Abtahi Froushani and Mashouri, 2018). Intrarectal administration of acetic acid can induce severe diffuse inflammatory reactions, leading ulcerations and erosions of the colon with profound leukocyte migration into the colon, particularly neutrophils (Abtahi Froushani and Mashouri, 2018; Sanei et al., 2014; Tahan et al., 2011). Myeloperoxidase (MPO) is a famous peroxidase enzyme mainly constituting the azurophilic granules of polymorphonuclear cells, which can be indirectly applied to estimate the intensity of neutrophil infiltration into a tissue specimen like colon in UC condition (Kondamudi et al., 2015). The inhibitory effect of thymol on the liberation of neutrophil elastase and leukocyte migration was reported previously (Braga et al., 2006). Here, we demonstrate that UC rats received thymol had lower MPO activity (as an indicator of neutrophil migration (in colonic homogenate compared to control UC rats. The hallmark of MPO activity is production of reactive oxygen species (ROS) by neutrophils (Winterbourn et al., 2016). Nitric oxide is another potentially harmful mediator that participating in the UC pathogenesis (Kolios et al., 2004). The uncontrolled or inappropriate production of reactive substances such as nitric oxide and ROS is involved in the prolongation and potentiation of inflammation (Motlagh et al., 2015). Interestingly, the nitric oxide level and malondialdehyde intensity were decreased in the colons of thymol treated rats more than prednisolone groups. The potent antioxidant activity of thymol is clear (Barbosa et al., 2017; Wang et al., 2017). This note somewhat verified the better results of thymol on reduction of myeloperoxidase and nitric oxide compared to prednisolone. In this regard, a former *in vitro *survey indicated that thymol could regress the declaration of inducible nitric oxide synthase (iNOS) in lipopolysaccharide primed mouse mammary epithelial cells (Xiao et al., 2015).

Clearly, the free radicals produced in the inflammatory condition like UC caused peroxidation of lipids in the colonic tissues (Liu et al., 2018; Rodrigues de Carvalho et al., 2018). The intensity of the lipid peroxidation may be measured by the level of malondialdehyde (Al-Rejaie et al., 2013b). It has been reported that thymol could significantly mitigate LPS-induced rise of MPO and MDA levels as well as the NF-κB expression in an acute lung injury mice model (Wan et al., 2018). The obtained data in our survey indicated that the intensity of MDA was suppressed in thymol treated class more profound than prednisolone received UC rats. Notably, unlike prednisolone, thymol possesses direct antioxidant benefits. The regression of MDA in prednisone received UC rats was consequentially flashed by inflammatory mediators.

Clearly, cytokines such as IL-1, TNF-α, and IL-6 play an essential function in the pathogenesis of UC (Liu et al., 2018). Interestingly, it has been documented that thymol can reduce the production of TNF-α, and IL-1β in lipopolysaccharide-treated macrophages via modulation of the expression of nuclear factors, including NFATs, JNK and AP-1(Gholijani et al., 2015). Pervious findings also noted that thymol could regress the ROS and TLR4-mediated NF-κB signaling pathways and MAPK signaling (Liang et al., 2014b; Wu et al., 2017). Here, we indicated that thymol could decrease the production intensity of IL-6, TNF-α and IL-1 in an in vivo scheme of inflammation.

Extensive infiltration of neutrophils and propagation inflammation interrupts the integrity of cells and mucosal improvement in the colonic tissues after UC induction (Al-Rejaie et al., 2013a). The current study showed that both medication could encourage the tissue healing since both can convert the intensity of reduction in total protein levels.

The damage induced by acetic acid after its luminal instillation causes the microflora to attack the lamina propria and intensify the inflammatory process (Fabia et al., 1993). Considerable scientific evidence indicated the direct antifungal and antibacterial properties of thymol (Marchese et al., 2016). It is, therefore, logical to consider some of the beneficial effects of thymol associated with its antimicrobial effect, along with its direct anti-inflammatory benefits.

Nowadays, anti-inflammatory medications such as glucocorticoids and 5-aminosalicylate are prescribed to control ulcerative colitis (Auphan et al., 1995; Joshi et al., 2005). This medication can inhibit both forms of cyclooxygenase isoenzymes (COX 1 and 2) that invoved in the propagation of inflammation (Consalvi et al., 2015). Targeting selectivity for COX-2 isoenzyme can lessen some of the adverse effects of these drugs like the risk of peptic ulceration (Consalvi et al., 2015). The attained data in this study, fortunately, indicated that thymol could suppress COX-2 activity in inflamed colon more than prednisolone. A decrease in the activation of SOCS3 can partially promote the suppression of COX-2 activity (Huang et al., 2012). In this regard, some findings suggested that thymol could inhibit expression of SOCS3 (Niu et al., 2015).

NF-κB, as a famous powerful pro-inflammatory transcription factor, can promote inflammatory processes. Moreover, many cytokines can lead to promotion of the NF-κB pathway (Li et al., 2016). The former reports showed that metformin and cavidine had medication potential in controlling the UC model induced by acetic acid via downregulation of the NF-κBp65 expression (Niu et al., 2015; Pandey et al., 2017). The obtained data in the current investigation emphasized that both thymol and prednisolone caused a significant regression in the mRNA expression of NF-κBp65 in the colitis rats. IκBα suppresses the NF-κB pathway by blocking nuclear localization signals. IκBα keeps NF-κB separated in an indolent position in the cytosol (Li et al., 2016). The analysis of mRNA level of IκBα did not indicate any significant diversity between the groups.

Finally, it is necessary to mention that prednisolone possesses a vigorous immunosuppressive nature with frequent side effects (Auphan et al., 1995). Conversely, thymol is a safe natural phenol with considerable immunomodulatory benefits with additional effects such as antimicrobials and antioxidant properties (Gholijani and Amirghofran, 2016; Marchese et al., 2016; Wang et al., 2017). A novel document demonstrated that thymol could modulate destructive immune responses attributed to antigen-specific immune response in ovalbumin-immunized mice (Gholijani and Amirghofran, 2016).

As a result, these findings suggest that treatment with thymol as a natural product is a promising strategy to ameliorate the signs of inflammation in an animal model of ulcerative colitis. Thymol, in addition to its direct anti-inflammatory properties, may have antimicrobial and antioxidant properties. However, other mechanisms may be involved in this regard; therefore, further studies are needed.
